# The Effect of the Controlled Release of Platelet Lysate from PVA Nanomats on Keratinocytes, Endothelial Cells and Fibroblasts

**DOI:** 10.3390/nano11040995

**Published:** 2021-04-13

**Authors:** Elena Filova, Andreu Blanquer, Jarmila Knitlova, Martin Plencner, Vera Jencova, Barbora Koprivova, Maxim Lisnenko, Eva Kuzelova Kostakova, Renata Prochazkova, Lucie Bacakova

**Affiliations:** 1 Department of Biomaterials and Tissue Engineering, Institute of Physiology of the Czech Academy of Sciences, 1083, 142 20 Prague, Czech Republic; Andreu.BlanquerJerez@fgu.cas.cz (A.B.); jarmila.knitlova@fgu.cas.cz (J.K.); martin.plencner@gmail.com (M.P.); lucie.bacakova@fgu.cas.cz (L.B.); 2 Department of Chemistry, Faculty of Science, Humanities and Education, Technical University of Liberec, Studentska 1402/2, 461 17 Liberec, Czech Republic; vera.jencova@tul.cz (V.J.); barbora.koprivova@tul.cz (B.K.); maxim.lisnenko@tul.cz (M.L.); eva.kostakova@tul.cz (E.K.K.); 3 Regional Hospital Liberec, Husova 357/10, 460 63 Liberec, Czech Republic; renata.prochazkova@nemlib.cz; 4 Faculty of Health Studies, Technical University of Liberec, Studentska 1402/2, 461 17 Liberec, Czech Republic

**Keywords:** controlled release, platelet lysate, PVA nanofibers, endothelial cells, keratinocytes, fibroblasts, cell differentiation

## Abstract

Platelet lysate (PL) provides a natural source of growth factors and other bioactive molecules, and the local controlled release of these bioactive PL components is capable of improving the healing of chronic wounds. Therefore, we prepared composite nanofibrous meshes via the needleless electrospinning technique using poly(vinyl alcohol) (PVA) with a high molecular weight and with a high degree of hydrolysis with the incorporated PL (10% *w/w*). The morphology, wettability and protein release from the nanofibers was then assessed from the resulting composite PVA–PL nanomats. The bioactivity of the PVA–PL nanomats was proved in vitro using HaCaT keratinocytes, human saphenous endothelial cells (HSVECs) and 3T3 fibroblasts. The PVA–PL supported cell adhesion, proliferation, and viability. The improved phenotypic maturation of the HaCaT cells due to the PVA–PL was manifested via the formation of intermediate filaments positive for cytokeratin 10. The PVA–PL enhanced both the synthesis of the von Willebrand factor via HSVECs and HSVECs chemotaxis through membranes with 8 µm-sized pores. These results indicated the favorable effects of the PVA–PL nanomats on the three cell types involved in the wound healing process, and established PVA–PL nanomats as a promising candidate for further evaluation with respect to in vivo experiments.

## 1. Introduction

Chronic wounds comprise a major health care issue, especially for patients with intercurrent illnesses and conditions such as diabetes, obesity and vascular disease. Chronic wounds are defined as wounds that either do not heal, or require long periods of time to heal, and which often reoccur after healing [1]. Chronic wounds comprise a wide range of wounds such as pressure ulcers, venous leg ulcers, arterial ulcers, neurotrophic ulcers, and foot ulcers in persons with diabetes. Despite their differing origins, all chronic wounds feature the presence of bacterial infection, persistent inflammation, elevated matrix metalloproteinase (MMP) production, the insufficient production of tissue inhibitors of metalloproteinases (TIMPs), enhanced matrix degradation, excessive proteolysis, growth factor degradation, lower cell proliferation levels, cell senescence, cell apoptosis and impaired angiogenesis/neovascularization [2]. Impaired vascularization leads to low oxygen tension, the insufficient supply of nutrients, further wound bed mutilation and delayed healing. The management of chronic wounds involves the treatment of the primary causes thereof via, e.g., vascular surgery, glycemic control, the treatment of infection, compression therapy, off-loading, the removal of necrotic tissue, etc.; however, some wounds persist for months or even years. In contrast to normal acute wounds, with respect to chronic ulcer edges, keratinocytes proved to be highly proliferative; they produced keratin 16 present in the activated phenotype, and did not produce any of the keratins involved in epidermal differentiation (K10, K2). In addition, the expression of the precursor of the α3 chain of laminin 5 (LM-3A32), involved in keratinocyte migration, was observed to be reduced [3]. Other studies [4,5] correlated increased levels of advanced glycation end products in diabetic patients, who often suffer from chronic wounds, with a lower proliferation of fibroblasts, the induced apoptosis thereof, and impaired nitric oxide (NO) and vascular endothelial growth factor (VEGF) production. Diabetic patients were also observed to evince a higher expression of MMPs, which acted to degrade the extracellular matrix. Moreover, endothelial cells in diabetic patients evince reduced NO production, increased reactive oxygen species (ROS) levels, and the altered expression of chemokines, growth factors and receptors for these factors [6]. High ROS levels lead to impaired neovascularization by influencing endothelial cell proliferation, migration and apoptosis. Therefore, efforts have been made to influence the treatment of chronic wounds at the cellular level via the delivery of growth factors and other bioactive compounds from various platelet-rich plasma (PRP) formulations. 

Platelets are involved in both the coagulation and inflammatory phases of acute wound healing. They produce a range of bioactive compounds, including platelet-derived growth factor (PDGF), transforming growth factors (TGF) from the α and β families, *fibroblast growth factor 2* (FGF-2), epidermal growth factor (EGF), vascular endothelial growth factor (VEGF), insulin growth factor-1 (IGF-1), stromal cell derived factor-1 (SDF-1), bone morphogenetic protein-2 (BMP-2), BMP-6, tumor necrosis factor-α (TNF-α) and platelet-activating factor (PAF). In addition, platelets release coagulation proteins, adhesive molecules, angiogenic factors, acidic hydrolases, cathepsin D, cathepsin E, thrombospondin-1, fibronectin and vitronectin, plasmin, TIMPs, MMP-2, MMP-9, inhibitors of proteases, bactericidal proteins, and the complement system [7,8,9,10,11,12]. Platelets are capable of interacting with leukocytes, regulatory T-cells, monocytes and endothelial cells in order to migrate, secrete pro-inflammatory cytokines and suppress inflammation via the regulation of macrophages; moreover, they interact with the complement system [13]. 

With regard to the improvement of the wound healing process, various platelet concentrates with platelet concentrations higher than that of the baseline have been evaluated, such as platelet-rich plasma (PRP), platelet gel, platelet-rich fibrin (PRF), serum eye drops (E-S) and PRP eye drops (E- PRP) [14]. A platelet-rich collagen gel that was successfully used in the treatment of chronic diabetic foot ulcers led to the complete epithelialization of the wounds of 17 out of 21 platelet-rich collagen gel-treated patients, compared to 2 out of 13 patients treated with a placebo after 8.6 weeks [15]. A randomized trial [16] assessed the effect of PRP gel on the healing of clean non-healing diabetic foot ulcers in 24 patients. The patients treated with the PRP gel were observed to require a shorter time to attain the maximal healing effect with the lowest resulting wound size (6.3 ± 2.1 vs. 10.4 ± 1.7 weeks). Moreover, only 8.3% of the patients subjected to PRP gel treatment failed to respond, compared to 41.6% of the patients treated with a control saline dressing. A further study [17] revealed that the treatment of chronic wounds of differing etiologies with platelet gel once per week in 24 patients resulted in complete healing in nine patients and a partial response in a further nine patients; the therapy of the other patients was terminated or they received cutaneous grafts. The platelet gel supported the formation of granulation tissue and re-epithelialization, and exerted an analgetic effect in all the patients [17].

A single-arm clinical trial, which involved 70 patients with chronic ulcers, analyzed the effect of platelet-rich plasma gel over 4 weeks on chronic ulcer healing [18]. After one month, the average wound area was found to have been reduced by 51%. The use of autologous platelet-rich plasma in diabetic patients with foot ulcers improved the healing process in comparison with standard therapy. However, in other types of wound, e.g., venous leg ulcers, arterious ulcers and pressure ulcers, the improvement was not evident [2]. A further study involved the use of a bio-functionalized scaffold composed of PRP and hyaluronic acid (HA) in the treatment of 182 patients with chronic ulcers (diabetic and vascular); HA alone was used as the control in 182 patients. The patients who underwent the combined treatment (PRP + HA) evinced 96.8% ± 1.5% re-epithelialization after 30 days compared to 78.4% ± 4.4% in the control group. The PRP/HA scaffold supported epidermal proliferation and dermal renewal to a greater extent than HA alone [19]. 

Platelet-based formulations have been used in various applications. For example, a platelet gel evinced the successful restitutio ad integrum of osteoradionecrosis following repeated administration and surgery in a seriously ill patient [20]. Autologous leukocyte- and platelet-rich plasma was applied to a burn scar during the first 5 consecutive days and at 30, 60, 90, 120 and 180 days. The treatment reduced the scar size and enhanced its maturation, texture, vascularity, pliability, induration and pigmentation [21]. An autologous platelet concentrate in combination with split thickness skin grafting (APC+STSG) and STSG alone was administered to grafted areas following the surgical treatment of 38 deep burns in 23 patients. APC+STSG was observed to enhance the recovery of the viscoelastic properties of the scars to a greater extent than STSG alone [22].

PRP has been incorporated into collagen gel [15] and into the scaffold composed of PRP and hyaluronic acid [19], via the incorporation of granules of lyophilized platelet-rich fibrin into a poly(vinyl alcohol) (PVA) hydrogel [23], via the binding of platelet-rich plasma [24] and the growth factors of interest [25] onto chemically modified or activated polycaprolactone (PCL) nanofibers, or via platelet lysate incorporation into the fibrin layer created on the surface of an electrospun nanofibrous blend of poly(L-lactide-*co*-ε-caprolactone) and poly(ε-caprolactone) [26].

This study involved the incorporation of platelet lysate (PL) into electrospun PVA nanofibers in order to fabricate a bioactive nanofibrous drug-releasing membrane as a potential skin wound dressing. Needleless electrospinning technology was used to form the PVA nanofibers. The PL was added to a water-based PVA spinning solution prior to electrospinning. The effect of the composite PVA–PL nanomats was evaluated in vitro using an HaCaT keratinocyte cell line, human saphenous endothelial cells (HSVECs) and a 3T3 fibroblast cell line so as to assess their potential with respect to enhancing the wound healing process. The effect of the PL-loaded PVA nanomats was compared with that of pure PVA nanomats and with PL that was added directly to the cell culture medium.

## 2. Materials and Methods

### 2.1. Platelet Lysate Preparation

A platelet suspension was prepared at the Transfusion Department of the Liberec Regional Hospital (Liberec, Czech Republic) from buffy coats obtained from 4 patients. Following collection, the blood was incubated at room temperature (RT) for one hour, and subsequently centrifuged at 3250 rpm for 14 min and processed in a blood-press apparatus. Following agitation at 22 °C for 30 min, the buffy coats from the 4 patients were mixed in a mixing bag under sterile conditions and supplemented with 300 mL of an additive solution (Composol PS, Fresenius Kabi, Bad Homburg, Germany), centrifuged at 1360 rpm (22 °C, 7 min), then processed in a Compomat G5 automatic press device. The leukocytes were removed by means of filtration (the resulting leukocyte content was lower than a 1 × 10^6^/transfusion dose). The final platelet concentration in the platelet suspension was determined to be in the range 600 × 10^9^ to 850 × 10^9^ platelets/L. The platelet suspension was frozen at −80 °C, following which it was slowly thawed (5 °C; overnight). After thawing, the platelet suspension was centrifuged at 2830× *g* (5 °C, 30 min). The supernatant, i.e., the platelet lysate (PL), was used either immediately or stored at −80 °C and thawed to 4 °C prior to use. The concentrations of fibroblast growth factor-2 (FGF-2), vascular endothelial factor (VEGF) and platelet-derived growth factor-BB (PDGF-BB) were measured using the standard manufacturers’ protocols for the following ELISA kits: FGF Human ELISA Kit (Cat. No KHG0021), VEGF Human ELISA Kit (Cat. No. KHG0111), PDGF-BB Human ELISA Kit (Cat. No. BMS2071), all of which were purchased from Thermo Fisher Scientific.

### 2.2. Nanofiber Preparation and Morphology Characterization

PVA with a molecular weight of 125,000 and degree of hydrolysis of 98–98.8% (Merck) was electrospun from a 10% (*w/w*) polymer solution in a water/ethanol solvent system (8/2, *w/w*). The PVA–PL was prepared from the same PVA; the PL was added to the spinning solution immediately prior to electrospinning as part of the aqueous portion of the solvent up to a final concentration of 10% *w*/*w* of PL in the spinning solution. A planar fibrous layer was prepared employing needleless electrospinning technology using a Nanospider™ NS 1WS500U device (Elmarco, Czech Republic). The 0.4 mm string and 0.6 mm slots were applied. The fibers thus formed were collected on a spunbond layer (polypropylene spunbond microfiber nonwoven substrate, Pegatex S; 20 g/m^2^; fiber diameter 20 µm; PFNonwovens Czech, Znojmo, Czech Republic) which was placed 14.4 cm above the string and rolled at a speed of 14 mm/min. Voltages of −10 kV and + 55 kV were applied to the collector and the string, respectively. The temperature was 21.5 °C and the relative humidity 25%. The electrospinning productivity of the PVA nanomaterial was 1.8 g per hour, and in the case of PVA with incorporated proteins, 2.8 g per hour. Typically, 10–20 g were prepared. With respect to the morphology evaluation, the samples were observed using a scanning electron microscope (SEM) with a Tescan Vega3 SB Easy Probe (Tescan, Brno, Czech Republic). The samples were sputter-coated with gold (14 nm) prior to the analysis. The morphological analysis of the SEM images was performed using NIS Elements software (Nikon). The diameter of the fibers was evaluated from at least 100 measurements. The PVA and PVA–PL nanofibers were sterilized by means of ethylene oxide at RT and stored for 14 days at 2–6 °C in a refrigerator.

### 2.3. Wettability of the Nanomats 

The wettability of the PVA-based electrospun nanofibrous materials with and without the addition of PL was tested using a Krüss K121 (Krüss GmbH, Hamburg, Germany) microtensiometer. A sample with a width of 30 mm and length of 40 mm was cut from the nanofibrous membrane and placed in a holder for foils, which was then inserted into a clip with the bottom edge of the sample arranged in the horizontal position during immersion in the test liquid (phosphate-buffered saline, PBS). The structure of the simple nanofibrous layer alone did not allow for the testing of wettability since a gel barrier formed during the measurements and, subsequently, the sample became twisted and was torn from the liquid. Thus, a model was created for the testing of the wicking of the PBS into the nanofibrous material on a polypropylene spunbond microfiber nonwoven substrate. The wicking, i.e., the weights of the PBS that wicked into the fibrous system and into the PVA on the spunbond microfiber over time, the PVA with platelet lysate (PVA–PL) on the spunbond microfiber, and the spunbond microfiber itself, were all measured three times. The spontaneous wicking of porous materials, including textiles and nanofibrous layers, can be described via the application of the Washburn equation [27,28]. The results, given in the form of the average squared mass gain over time from the beginning of the wicking process, represented the wicking rate via the direction of the regression curve.

### 2.4. Protein Release from the PVA–PL Nanomats

PVA–PL samples of 20 mg in weight were incubated in 2 mL of PBS at 37 °C. The release of total protein into the buffer was quantified after 5, 10 and 20 min, 1, 2 and 4 h, and 1, 3 and 7 days of incubation. At each time point, 200 µL of the solution was extracted and 200 µL of fresh PBS was added. The protein content was measured using a Micro BCA Protein Assay kit (Thermo Fisher Scientific, Waltham, MA, USA) according to the manufacturer’s instructions. The release of the protein was calculated both as its release between two time points and its cumulative release over seven days from 7 parallel samples.

### 2.5. Cell Models and Culture Conditions

A HaCaT human keratinocyte cell line (CLS Cell Lines Service GmbH, Eppelheim, Germany) was expanded in Dulbecco’s modified Eagle medium (DMEM, Life Technologies Czech Republic s.r.o., Prague, Czech Republic) with 10% fetal bovine serum (FS) and gentamycin (40 µg/mL, LEK Lek Pharmaceuticals d.d., Ljubljana, Slovenia). The cells were seeded at a density of 5000 cells/well in 1 mL in a 24-well glass bottom cell culture plate (Cellvis, Sunnyvale, CA, USA) or on glass coverslips inserted into a 24-well cell culture plate (TPP, Trasadinen, Switzerland). The cells were cultured in the medium supplemented with 2% of FS and with 1, 2.5 and 5% PL, PVA (1 cm^2^) or PVA–PL (1 cm^2^) nanofibers, respecitvely, for 7 and 14 days. 

Primary HSVECs (Provitro AG, Berlin, Germany) were expanded in Endothelial Cell Growth Medium 2 (EGM 2) with supplements (EGM-full, PromoCell GmbH, Heidelberg, Germany Cat. No. C-22111). This medium was used as a positive control for the optimization of the PL concentration in an EGM-weak (EGMw) HSVEC culture medium. The EGMw medium was prepared from the EGM 2 medium by adding 1% of antibiotic-antimycotic solution (Sigma), FS, heparin, ascorbic acid and hydrocortisone; however, all the growth factors were excluded from the supplements. The HSVEC seeding density was 20,000 cells/well in 1 mL in a 24-well cell culture plate (TPP, Trasadinen, Switzerland; or Cellvis, USA). 

The seeding density of mouse 3T3-Swiss albino (ATCC^®^CCL-92^™^) fibroblasts (Chemos CZ, s.r.o.) was 5000 cells/well in 1 mL of DMEM with 2% of FS or of DMEM with 10% FS (control sample) in a 24-well plate (TPP, Switzerland) or a high-performance 24-well glass bottom plate (Cellvis, U.S.A, BIO-PORT Europe s.r.o.). The PVA and PVA–PL nanomats were added to the medium 1 h following cell seeding and floated above the cells during the whole of the cultivation period.

### 2.6. Cell Morphology, Cell Viability

The cell viability was measured using a CellTiter 96^®^ AQ_ueous_ One Solution Cell Proliferation Assay (MTS, Promega, Madison, WI, USA). On days 1, 3, 7 and 14 the cell culture medium in the wells with the samples was replaced with 500 µL of DMEM supplemented with 2% of FS, and 100 µL of MTS reagent was subsequently added to the medium. The samples were incubated for 1 h and the absorbance was measured at 490 nm. The medium without cells with the added reagent was used as the negative control. In total, 3–4 samples were evaluated for each experimental group and time interval. 

### 2.7. Cell Visualization, the Immunofluorescence Staining of the Cell Differentiation Markers, and Cell Population Density

The cells were visualized via staining with Texas Red C_2_ Maleimide, which acts to bind compounds with thiol groups in the cells, or via staining with phalloidin, which binds filamentous actin (F-actin). The Texas Red staining was applied to the HaCaT cells and 3T3 fibroblasts for the cell population density evaluation. These cells were fixed with 4% paraformaldehyde, permeabilized in PBS containing 1% bovine serum albumin (BSA) and 0.1% Triton X-100 for 20 min, washed with PBS and incubated with a Texas Red C_2_-Maleimide solution (6.86 × 10^−4^ M, Thermo Fisher Scientific) for 1 h at RT, and subsequently washed twice with PBS. The phalloidin staining was applied to the 3T3 fibroblasts and HSVECs. Following fixation and permeabilization, these cells were incubated with a phalloidin–tetramethylrhodamin (TRITC) conjugate (2 µg/mL, Sigma-Aldrich, Cat. No. P1951) for 1 h at RT. The cells were then washed twice with PBS. 

The development of the intermediate filaments on the keratinocytes was evaluated via the immunodetection of basal cytokeratins, which involved cytokeratins 5 and 14, which are associated with the proliferative state, and the detection of cytokeratin 10, which is associated with the more differentiated level of keratinocytes in the epidermal layers. The HaCaT cells were cultured on glass coverslips for 3 and 14 days. The HSVECs were cultured for 3 and 7 days and their maturation status was assessed via the immunofluorescence staining of the von Willebrand factor. Vinculin, a focal adhesion plaque protein associated with integrin adhesion receptors, and type I collagen, an important extracellular matrix (ECM) protein produced by fibroblasts, were visualized in the 3T3 fibroblasts via immunofluorescence staining on days 1 and 7, respectively. 

With respect to the immunofluorescence staining, the cells were fixed in 4% paraformaldehyde in PBS for 15 min at RT, washed twice with PBS and permeabilized with 1% BSA in PBS containing 0.1% Triton X-100 for 20 min. They were then washed with PBS, treated with 1% Tween for 20 min at RT and washed again with PBS. The samples seeded with the HaCaT keratinocytes were fixed on day 3 and incubated with mouse anti-basal cell cytokeratin (clone RCK103, MUbio BV; dilution 1:200 in PBS) overnight at 4 °C. The samples fixed on day 14 were incubated with the rabbit monoclonal anti-cytokeratin 10 antibody (Abcam; dilution 1:400) overnight at 4 °C and, following rinsing twice with PBS, some of the samples were incubated with the mouse monoclonal anti-cytokeratin 14 antibody (Abcam; dilution 1:400) for 3 h at RT. The samples seeded with the HSVECs were stained using a rabbit-produced anti-von Willebrand factor antibody (Merck, Cat. No. F3520; dilution 1:200) at 4 °C overnight and washed twice with PBS. The samples seeded with the 3T3 fibroblasts were incubated with a mouse-produced anti-vinculin antibody (Sigma, Cat. No. V9131; dilution 1:200) or with a rabbit-produced anti-type I collagen antibody (Cosmo Bio, Cat. No. LSL-LB-1197, dilution 1:200) at 4 °C overnight. 

After being washed twice in PBS, the samples were incubated with an anti-mouse secondary antibody conjugated with Alexa Fluor488 (Thermo Fisher Scientific, A11017, 1:400), an anti-rabbit secondary antibody conjugated with Alexa Fluor 488 (Thermo Fisher Scientific A11070, 1:400), and a goat anti-mouse secondary antibody conjugated with Alexa Fluor 546 (Thermo Fisher Scientific, A11003, 1:400). The antibodies were diluted in PBS with Hoechst 33,258 (0.020 mM) and each antibody was applied for 1 h at RT. After washing twice in PBS, the samples were observed under a Leica SPE confocal microscope or an IX71 Olympus epifluorescence microscope equipped with a D71 digital camera. All the images taken following the staining of the cells (i.e., with Texas Red C_2_ Maleimide, phalloidin-TRITC or immunofluorescence staining) were used for the counting of the cells and the calculation of the cell population densities. The intensity of immunofluorescence staining for von Willebrand factor in HSVECs was measured on micrographs (7–10 measurements per each group) using the ImageJ software and was normalized per cell.

### 2.8. Transmigration Assay 

FluoroBlok cell culture inserts with pores of 8 µm in diameter (Corning, Cat No. 351152) were placed in each well of a 24-well companion plate with a low evaporation lid (Thermo Fisher Scientific, Cat. No. 9993.9150). In total, 750 µL of pure EGMw medium, EGMw medium supplemented with 1, 2.5 and 5% of PL, EGMw medium with PVA nanofibers or with PVA–PL nanofibers or, alternatively, EGM-full medium were placed in the bottoms of the wells (*n* = 3). The HSVECs were seeded at a density of 20,000 cells/500 µL of EGMw medium in all the inserts. After 4 h in the culture, the inserts were washed with PBS and fixed in 4% paraformaldehyde in PBS for 15 min at RT. The cells were then stained with a combination of Texas Red C_2_-Maleimide dye (1.7 µg/mL, Thermo Fisher Scientific) and Hoechst 33,258 nuclear dye (0.020 mM, Sigma-Aldrich spol. s.r.o., Prague, Czech Republic) for 1 h at RT. Images of the cells that crossed the insert membranes were captured under an Olympus IX71 epifluorescence microscope equipped with a DP80 digital camera (both from Olympus, Tokyo, Japan). 

### 2.9. Statistical Evaluation

The One-Way Analysis of Variance (ANOVA) Student–Newman–Keuls multiple comparison test was used for the statistical evaluation; a *p* value ≤ 0.05 was considered significant. 

## 3. Results and Discussion

### 3.1. Preparation and Characterisation of the PVA Nanomats 

PVA is a biocompatible polymer which is highly hydrophilic and chemically resistant; it exhibits good mechanical properties, is non-degradable under most physiological conditions [29], and can be processed into gels, foils or nanofibers. With concern to wound dressing applications, skin regeneration and skin tissue engineering, PVA has been used alone or in combination with other natural and synthetic polymers, e.g., dextran, starch, alginate, chitosan, glucan, gelatin, polylactide (PLA) and poly(N-vinylpyrrolidone). The solubility of PVA is reduced via chemical crosslinking, γ-irradiation and freeze–thawing cycles [30]. Due to the fact that PVA is a water-soluble polymer, it is suitable for the incorporation of biologically active substances including proteins. Subsequent crosslinking, usually performed chemically, at high temperatures or via UV-radiation, is necessary in order to retard the release of incorporated substances. These procedures often lead to the cytotoxicity of the resulting material or a reduction in the activities of incorporated substances [31]. In the case of PVA, however, it is possible to make use of its natural ability to create a physical network. In addition to the molecular weight, the degree of hydrolysis comprises an important parameter with respect to this polymer. With an increasing degree of hydrolysis, the solubility of PVA in aqueous environments decreases due to the formation of strong non-covalent interactions, i.e., hydrogen bonds between the hydroxyl groups and Van der Waals interactions between the hydrocarbon polymer backbones [32]. This, in turn, affects, inter alia, the release of incorporated substances—in our case, proteins from the platelet lysate [33].

PVA with a degree of hydrolysis of 98–98.8% and a molecular weight of 125,000 was used in this study for the preparation of the nanofibrous layers. The PVA nanofibers and the PVA–PL nanofibers were prepared via the electrospinning of a PVA solution in water/ethanol or in a water/PL/ethanol solvent. As determined by previous measurements, the platelet lysate used in the preparation of the PVA–PL contained 16.4 ± 0.7 ng/mL of PDGF-BB, 62.9 ± 5 pg/mL of FGF-2 and 51.2 ± 0.7 pg/mL of VEGF. Another study [34] confirmed the presence of various growth factors in the platelet lysate (900 × 10^9^ platelets/mL), such as TGF-β1, PDGF-BB, VEGF, EGF, HGF, FGF-2, IGF-1, KGF and SDF-1, as well as G-CSF, GM-CSF, and other cytokines and chemokines. Growth factors are of key importance in terms of modulating the cell response during healing. A reasonably high content of 10% PL in the spinning solution assured the homogeneity of the prepared PVA–PL fibers. 

**Figure 1:**
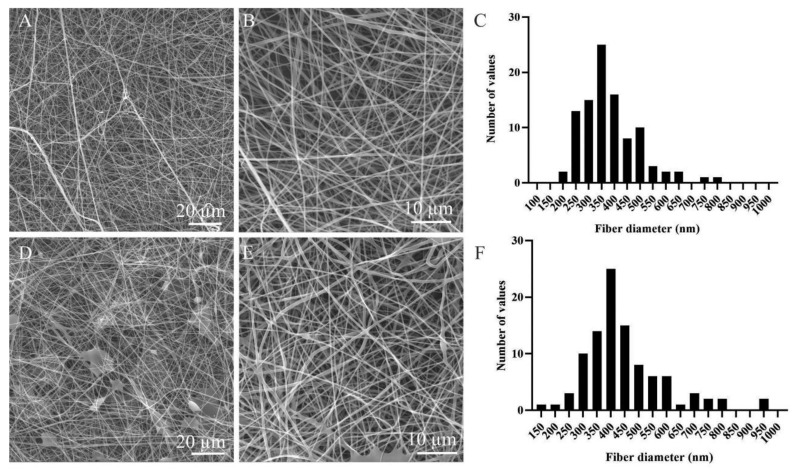
Scanning electron microscope (SEM) images of the electrospun poly(vinyl alcohol) (PVA) (**A**,**B**) and PVA–platelet lysate (PL) (**D**,**E**), magnification × 2000 (scale bar = 20 µm) and ×5000 (scale bar = 10 µm), respectively. Histograms of the fiber diameter distributions of the PVA (**C**) and PVA–PL (**F**) nanomats.

The SEM images revealed the presence of homogeneous nanofibrous layers (Figure 1A,B,D,E); the distribution of the fiber diameters is shown in the graphs in Figure 1C,F. The surface density was determined at 4.3 ± 1.17 g/m^2^ for the PVA and 6.75 ± 1.85 g/m^2^ for the PVA–PL (mean ± SD). The diameter of the nanofibers was 0.38 ± 0.17 µm for the PVA and 0.37 ± 0.15 µm for the PVA–PL (mean ± SD). Interestingly, the formation of fibers was improved by adding PL to the PVA spinning solution. However, the dissolution rate of the PVA–PL fibers in a water-based environment was observed to be more rapid than that of the pure PVA [33]. 

**Figure 2:**
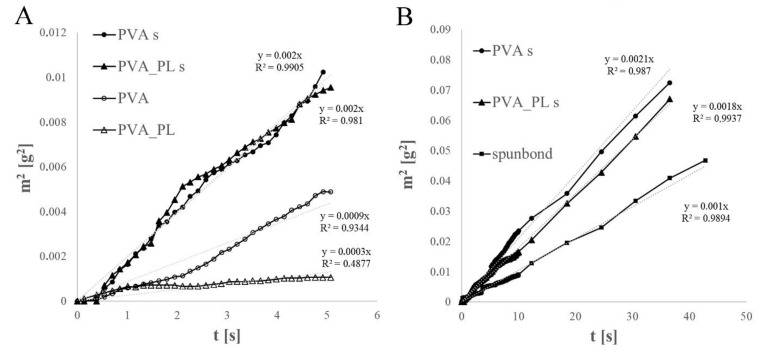
Mean square weight of the PVA, PVA–PL, spunbond, PVA on the spunbond (PVA s) and PVA–PL on the spunbond (PVA–PL s) at 0–5 s (**A**) and at 0–40 s (**B**) following the immersion of the nanofibers in PBS.

The PVA and PVA–PL nanofibers were also found to differ in terms of their wettability, which was measured via PBS penetration and wicking. The mean square weights of the PBS that penetrated into the self-standing and spunbond-supported PVA and PVA–PL electrospun materials during 5 s of wicking are presented in Figure 2A. The creation of a gel barrier decreased the rate of PBS penetration into the nanofibrous materials. The gel barrier was formed more rapidly with regard to the material with the platelet lysate, i.e., the rate of penetration was significantly lower. The comparison of the PVA and PVA–PL nanofibers electrospun onto the spunbond material over longer time periods (up to 35 s) evinced very similar degrees of PBS wicking into both the tested materials, although the PVA–PL demonstrated a slightly lower rate of spontaneous PBS penetration vertically upwards into the fibrous material (see Figure 2B).

**Figure 3:**
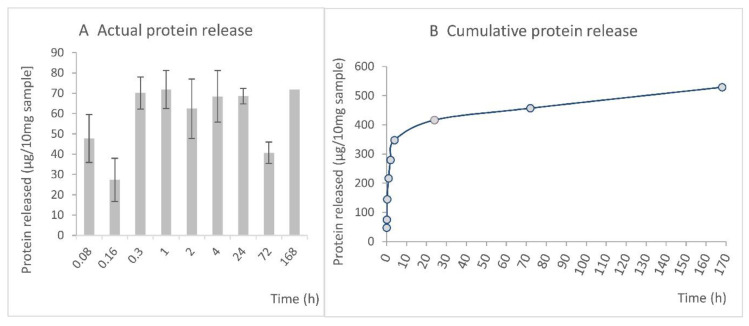
Actual protein concentration (**A**) and cumulative protein release (**B**) from the PVA–PL nanomats during a 7-day incubation in phosphate-buffered saline (PBS), data expressed as mean ± Standard Deviation (S.D.) (**A**), mean ± Standard Error of Mean (S.E.M.) (**B**).

Our study involved the use of PVA with a high molecular weight (125,000) and a high degree of poly(vinyl acetate) hydrolysis, i.e., in excess of 98%. PVA with a reduced degree of solubility allows for both the incorporation of PL and the prolonged release of PL from PVA–PL nanomats [33], as was proved by the measurement of the protein release curve. The rapid increase in the protein concentration following the immersion of the PVA–PL in PBS was characterized by an initial burst release during the first hour (Figure 3A), following which the concentration of proteins in the PBS appeared to remain stable until day 3, when it began to decrease. The cumulative release curve (Figure 3B) illustrates the burst release of the protein during the first 4–24 h, followed by its continuous release up to day seven. In addition to the release of protein, the decreased protein concentration values at later time intervals may also have been influenced by the degradation/denaturation of the proteins in the PBS buffer after 3 days. This PVA mesh growth factor release system closely resembles the real situation in wounds. 

### 3.2. Viability, Growth, Morphology and the Differentiation of Keratinocytes, Endothelial Cells and Fibroblasts

#### 3.2.1. The Effect of the PVA Nanomats and PL on HaCaT Keratinocytes

**Figure 4:**
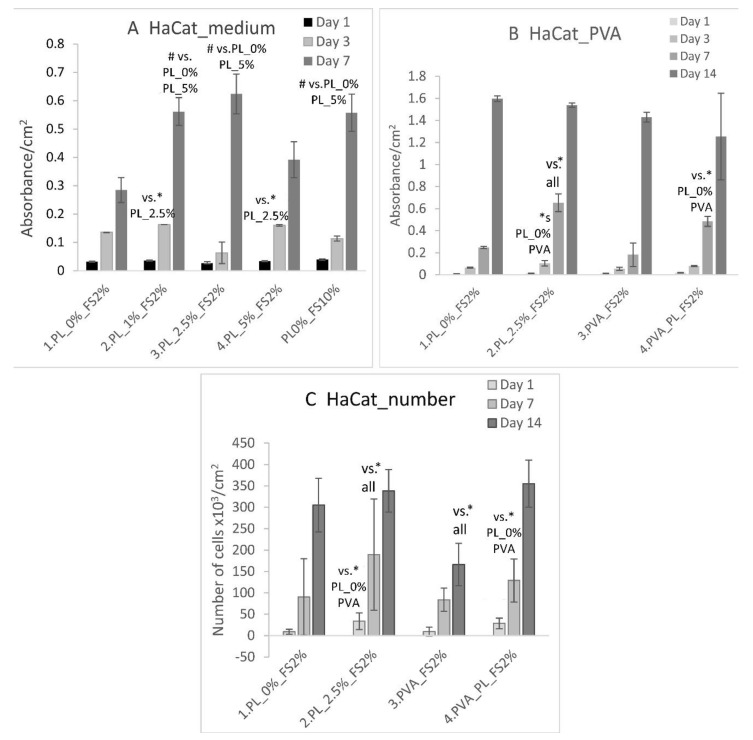
Viability of the HaCaT cells cultured on glass in Dulbecco’s modified Eagle medium (DMEM) supplemented with 2% fetal bovine serum (FS) and 0, 1, 2.5 and 5% of PL, and in DMEM supplemented with 10% of FS (**A**). The viability (**B**) and population density (**C**) of the HaCaT cells in DMEM supplemented with 2% of FS and 0 and 2.5% of PL, and in DMEM supplemented with 2% of FS and with the addition of PVA and PVA–PL nanomats. The cells were cultured for 1–7 days (**A**) and for 1–14 days (**B**,**C**). The data are presented as the mean ± S.D., # is used for *p* < 0.001, * for *p* < 0.05.

HaCaT cells were first cultured in DMEM with 2% FS and various PL contents for 7 days (Figure 4A). On day 1, the HaCaT cells displayed similar viability regardless of the cell culture medium applied. On day 3, a higher level of HaCaT cell viability was observed in the media containing 1% and 5% of PL than in the medium with 2.5% of PL. On day 7, the highest cell viability values were observed in the DMEM with 2.5% and 1% of PL and in the medium 

The DMEM with 2% FS and 2.5% PL was used as the positive control in further experiments with the PVA nanomats using HaCaT cells. The optimum PL concentration determined by our experiment was somewhat lower than the concentrations observed in other studies. For instance, Baik et al. studied the dependence of HaCaT proliferation on the PL preparation method, especially applying one to three freeze–thaw cycles and various platelet concentrations (1 × 10^12^/L and 2 × 10^12^/L). The optimum medium for HaCaT cell proliferation comprised 5% PL (2 × 10^12^/L platelets) with the application of two freeze–thaw cycles [35]. The platelet lysate with 2 × 10^12^/L platelets was found to contain high concentrations of growth factors, i.e., 8.5 ± 2.8 µg/mL of TGF-α, 488.9 ± 126.1 µg/mL of VEGF, 634.194 ± 81.184 µg/mL of PDGF-AB/BB, 80.148 ± 27.589 µg/mL of PDGF-AA and 1.301.5 ±142.1 µg/mL of EGF [35]. For the purpose of comparison, the PL used in our experiments evinced a ~10^7^ times lower concentration of VEGF and a ~39,000 times lower concentration of PDGF-BB. Thus, it appears that PL acts in a wide range of concentrations. A further study reported that a cell culture medium supplemented with 10% or 20% PL enhanced the closure of a scratched area by NCTC 2544 line dermal keratinocytes over 72 h [36]. The platelet concentration was 1.3 × 10^12^ platelets/L; a lower platelet concentration was, however, found to be ineffective. The concentration of PDGF-AB in the PL considered in the study was 42 ± 9 ng/mL, the concentration of TGF-β was 64 ± 5 ng/mL and the concentration of VEGF was 740 ± 110 pg/mL [36]. 

With respect to our experiment with PVA nanomats, the HaCaT cells evinced similar viability values and cell population densities for all the tested samples after 1 day of culturing (Figure 4B,C). On day 3, a significantly higher HaCaT cell viability was determined in the medium with 2.5% of PL than in all the other samples. On day 7, the highest HaCaT cell viability was observed, again, in the medium containing 2.5% of PL. However, the cells grown in the presence of the PVA–PL mats evinced a higher viability than the cells grown with the pure PVA mats or in the DMEM without PL. On day 14, similar HaCaT cell viability values were observed for all the tested samples. A similar trend was observed with respect to the cell population density. On day 1, HaCaT cells adhered in higher densities in both the medium with 2.5% PL and the medium with PVA–PL nanomats (Figure 4C). On day 7, cell population density was observed to have increased in the medium with 2.5% PL, although after 14 days, similar results were observed for all the samples except for the pure PVA, concerning which the final values were significantly lower (Figure 4C). Firstly, these results indicate that PL is more efficient during early culture intervals (up to day 7) than in later culture intervals (day 14), which corresponds to its relatively high and stable rate of release during the first three days of culturing (Figure 3). Secondly, it appears that the controlled release of platelet lysate from PVA–PL nanomats, although in small quantities, is only slightly less effective than when platelet lysate is added to the cell culture medium in the form of a single dose. PVA that has been subjected to the continuous dissolution and release of PL may also contribute to the favorable effect of PVA–PL meshes by preserving protein bioactivity and decreasing protein degradation.

**Figure 5:**
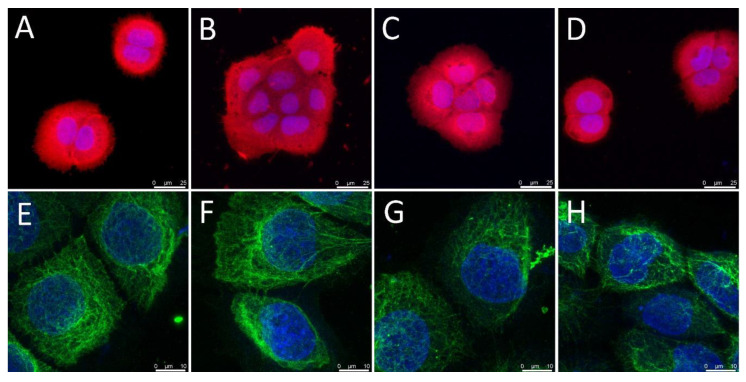
Texas Red C_2_-maleimide staining of the HaCaT cells on day 1 following seeding (**A**–**D**), and the immunofluorescence staining of the basal cytokeratins in these cells on day 3 following seeding (**E**–**H**). The cells were seeded on glass in DMEM containing 2% of FS without the presence of PL (**A**,**E**), with the presence of 2.5% of PL (**B**,**F**), with the presence of PVA nanomats (**C**,**G**) and with the presence of PVA–PL nanomats (**D**,**H**). The cell nuclei were counterstained with a Hoechst 33258. Leica SPE confocal microscope, scale bar = 25 and obj. × 63 (**A**–**D**), scale bar = 10 µm and obj. × 100 (**E**–**H**).

With respect to the cell morphology, the HaCaT cells adhered, spread and grew in clusters (i.e., islands) that are typical for keratinocytes from day 1 following seeding (Figure 5A–D). The cell clusters expanded over time and formed a confluent cell layer on day 14. On day 3, the HaCaT cells were positively stained for basal cytokeratins, which formed intermediate filaments typical of basal keratinocytes (Figure 5E–H). 

**Figure 6:**
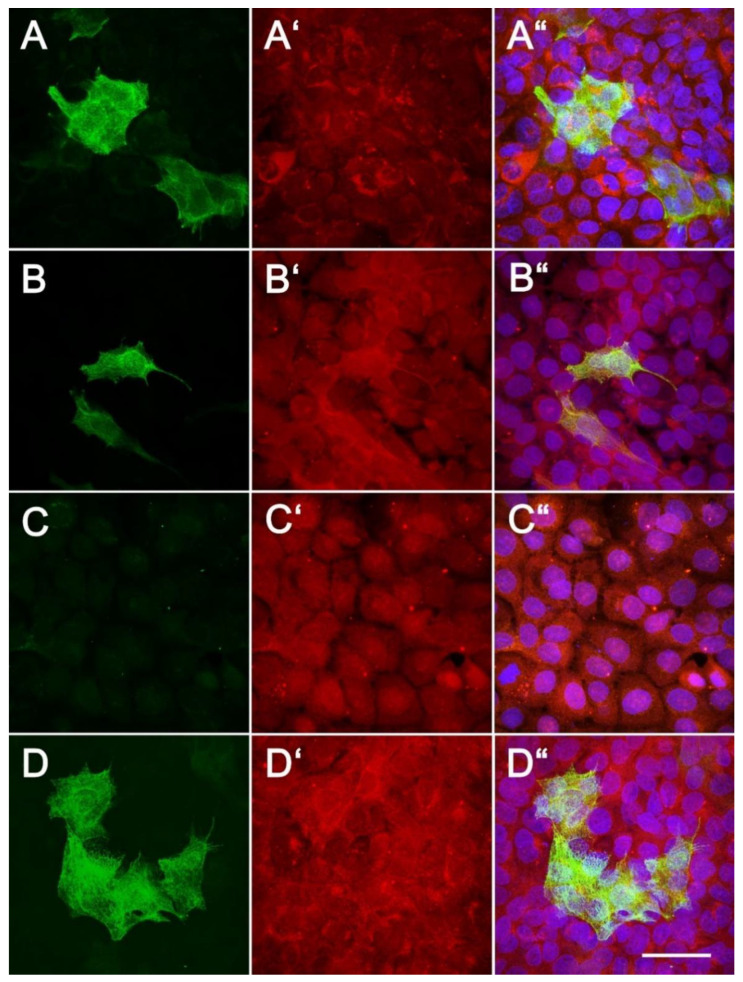
Immunofluorescence staining for cytokeratin 10 (green) (**A**,**A“**,**B**,**B“**,**C**,**C“**,**D**,**D“**) and cytokeratin 14 (red) (**A‘**,**A“**,**B‘**,**B“**,**C‘**,**C“**,**D‘**,**D“**) of the HaCaT cells following 14 days of culturing. The cell nuclei were counterstained with Hoechst 33258 (blue). The cells were grown on glass in DMEM containing 2% FS without the presence of PL (**A**,**A‘**,**A“**), in DMEM containing 2% FS and 2.5% PL (**B**,**B‘**,**B“**), in DMEM containing 2% FS with the presence of pure PVA (**C**,**C‘**,**C“**), and in DMEM containing 2% FS with the presence of PVA–PL (**D**,**D‘**,**D“**). Leica SPE confocal microscope, obj. × 63, scale bar = 25 µm.

On day 14, the HaCaT cells were found to be positive for cytokeratin 14 (Figure 6). Moreover, a second layer of HaCaT cells appeared in the media without PL, with 2.5% of PL and with the PVA–PL nanomats, which formed filaments positive for cytokeratin 10. Interestingly, no cytokeratin 10-positive cells were observed in the medium with the pure PVA (Figure 6C). Keratinocyte differentiation requires complex signals in their environment. HaCaT cells differentiate and create squamous epithelium when co-cultured with fibroblasts at a high population density (5 × 10^5^ cells/mL) in a collagen gel; however, their proliferation and differentiation is delayed by the lower population density of the fibroblasts (2 × 10^5^ cells/mL) [37]. The considerable deposition of laminin, nidogen and collagen IV was observed in the gels with higher densities of fibroblasts at 2 and 3 weeks following cell seeding. Similarly, basement membrane proteins (e.g., collagen VII, laminin V) have been observed to be deposited to an extent comparable to that in native skin in co-cultures of HaCaT cells and fibroblasts in collagen gels with 2 × 10^4^ and 8 × 10^4^ fibroblasts per mL [38].

#### 3.2.2. The Effect of the PVA Nanomats and PL on HSVEC Cells

**Figure 7:**
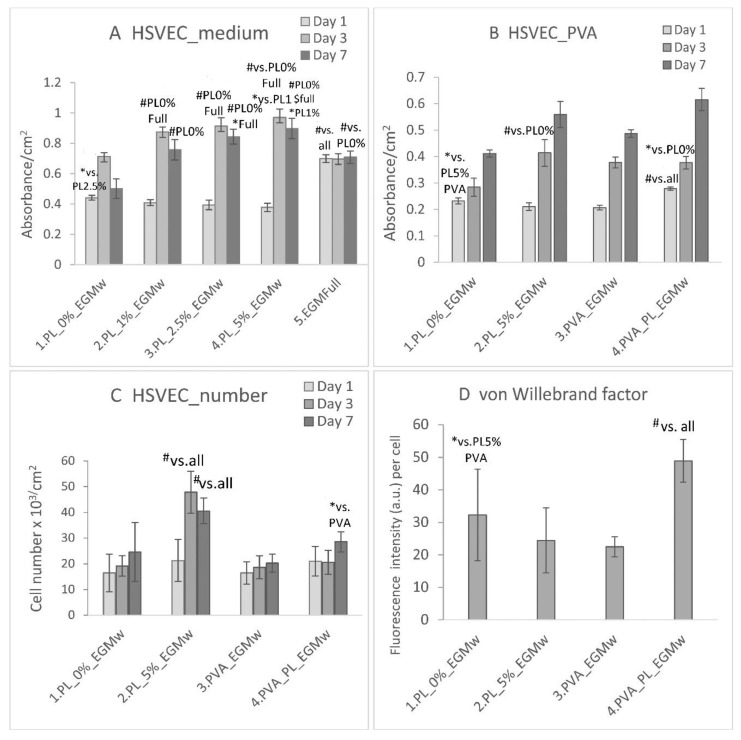
Viability/cm^2^ of the human saphenous endothelial cells (HSVECs) in the EGM-weak (EGMw) medium, the EGMw supplemented with 1, 2.5 and 5% PL and the EGM-full medium (**A**). Viability/cm^2^ (**B**) and population density/cm^2^ (**C**) of the HSVECs in the EGMw medium, the EGMw with 5% of PL, the EGMw with added PVA and PVA–PL nanomats and the EGM-full medium. The cells were cultivated for 1, 3 and 7 days (**A**,**B**). The intensity of fluorescence of the von Willebrand factor in the HSVECs on day 7 following seeding (**D**). The data are expressed as the mean ± SD, # refers to *p* < 0.001, $ to *p* < 0.01, * to *p* < 0.05.

HSVECs were cultured in the EGM 2 medium either with or without the recommended supplements. Aimed at optimizing the PL concentration for further experiments involving PVA nanomats, the HSVECs were cultured in the EGM 2 medium without the addition of growth factors such as IGF, EGF, VEGF and FGF, referred to as EGMw. On day 1 following seeding, the highest cell viability was observed in the EGM-full medium, which contained all the supplements (Figure 7A). Interestingly, a higher cell density was also determined in the EGMw without PL than in the samples in the medium with 2.5–5% of PL. 

On day 3, however, the medium with PL significantly increased the cell viability proportionally to the PL concentration in comparison with the values in both the EGMw and EGM-full samples. The highest value was obtained with respect to 5% PL. On day 7, the highest cell viability was observed in the EGMw with 5% and 2.5% PL. The cell viability was the same in the EGM-full medium for all the culture intervals. A possible explanation lies in the cells having attained confluence very rapidly, following which no considerable change was observed in their number. The EGMw with 5% of PL was selected as the positive control for further experiments with HSVECs in the presence of PVA nanomats.

**Figure 8:**
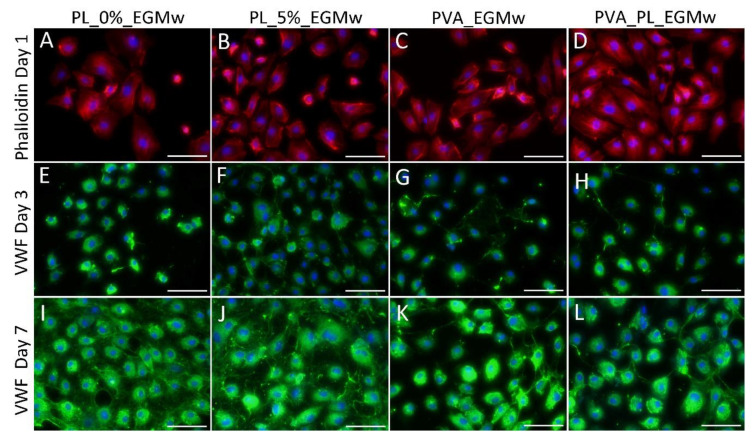
Staining of the HSVEC cells with phalloidin–tetramethylrhodamin (phalloidin-TRITC) for F-actin on day 1 following seeding (**A**–**D**) and the immunofluorescence staining of the von Willebrand factor (VWF) on day 3 (**E**–**H**) and on day 7 (**I**–**L**). The cells were seeded on glass in the EGMw medium without PL (**A**,**E**,**I**), with 5% of PL (**B**,**F**,**J**), with PVA (**C**,**G**,**K**) and with PVA–PL (**D**,**H**,**L**). Olympus epifluorescence microscope IX71, D71 digital camera, obj. × 20, scale bar = 100 µm.

The HSVECs spread in the EGMw with the PVA–PL and the PVA nanomats and evinced similar polygonal morphologies in all the samples on day 1 (Figure 8). The cell viability on day 1 was observed to be higher in the medium with the PVA–PL than in any of the other samples (Figure 7B). The lowest cell viability was observed in the EGMw with PVA on day 1. On the same day, the cell population density was found to be similar in all the samples (Figure 7C). On day 3 following seeding, the viability of the HSVECs increased to a greater extent in the EGMw with 5% PL, PVA and PVA–PL than in the pure EGMw. On the same day, the cell number increased in the EGMw with 5% PL compared to all the other samples. After 7 days, the viability of the HSVECs was observed to be similar for all the samples; however, the cell population density was highest in the EGMw with 5% PL. Moreover, the PVA–PL nanomats supported HSVEC growth to a greater extent than did the PVA when added to the EGMw medium.

The positive effect of platelet lysate on HSVEC viability might be explained by the presence of biomolecules that are important for endothelialization such as VEGF, FGF-2, EGF, sphingosine-1-phosphate (S1P), angiopoietin-1 (Ang1) and platelet-derived endothelial growth factor (PD-ECGF) [10,39,40]. Conversely, platelets also release certain anti-angiogenic factors, i.e., thrombospondin-1 (TSP-1), endostatin and platelet factor 4. A study by Romaldini et al. determined that while platelet lysate (5%) stimulated the proliferation of human umbilical vein endothelial cells, it attenuated the activation of the NF-κB pathway via IL-1 and maintained the ability of EC to form tube-like structures on Matrigel [41]. Cell culture media supplemented with 5% of a mixture of PL/platelet-poor plasma (PPP, 10^7^ platelets/mL) at various ratios support the proliferation of human bone marrow mesenchymal stromal cells (hMSCs), adipose tissue-derived MSCs, human lymphocytes and umbilical cord-derived MSCs more effectively than do media supplemented with 10% of fetal calf serum (FCS) or with 10% of FCS + 1 ng/mL of FGF-2 [42]. 

At the same time, PL/PPL mixtures support the formation of medium- and large-sized cell colonies, with the hMSCs maintaining their potential for differentiation into multiple cell types. Moreover, platelet lysate (5%) has been found to stimulate the differentiation of MSCs toward endothelial cells when exposed to fluid shear stress (2.5–10 dyn/cm^2^) [43].

While on day 3, the HSVECs were positively stained for the von Willebrand factor, the staining was stronger on day 7 (Figure 8). After 7 days culturing, the HSVECs evinced a cobblestone morphology, especially in the regions with confluence. The intensity of the fluorescence staining of the von Willebrand factor produced by the HSVECs as measured on day 7 was significantly greater in the medium with the PVA–PL than in any of the other samples, whereas it was slightly lower in the pure EGMw medium. The Von Willebrand factor is regarded as a marker of the phenotypic maturation of endothelial cells, and it is involved in hemostasis. It is produced by endothelial cells and megakaryocytes, and is stored in the Weibel–Palade bodies of endothelial cells or in the α-granules of platelets. Moreover, it is secreted from cells into the ECM and the blood [44]. 

**Figure 9:**
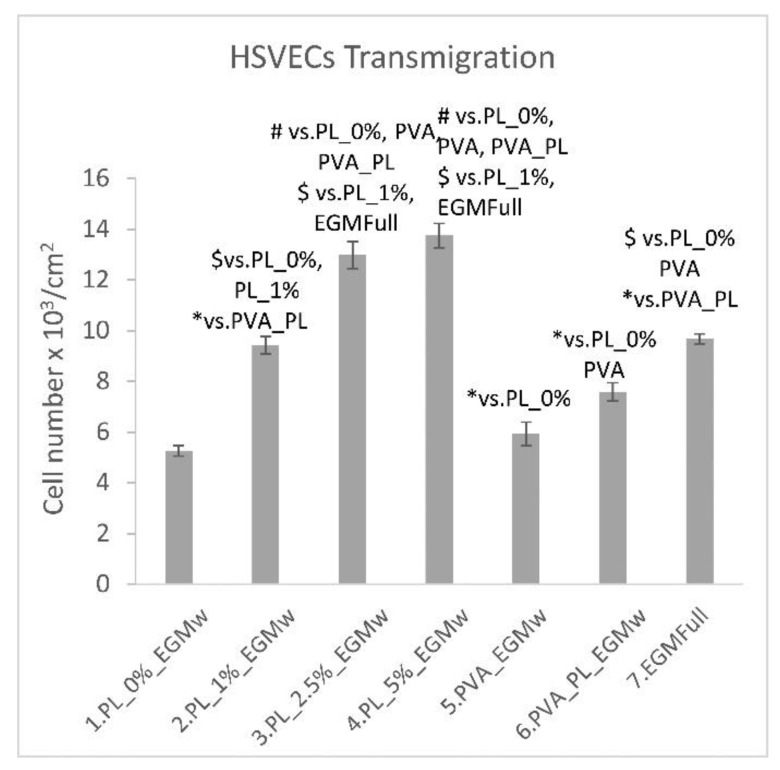
Transmigration assay of the HSVECs toward the EGMw medium, and the EGMw supplemented with 1, 2.5 and 5% of PL, with PVA nanofibers, and with PVA–PL nanofibers, and toward the EGM-full medium at 4 h following seeding. The data are presented as the mean ± S.D. from 3 samples, # refers to *p* < 0.001, $ to *p* < 0.01, * to *p* < 0.05.

All the tested concentrations of PL acted as a chemoattractant that led to HSVEC migration through the pores in the cell culture inserts towards the medium containing the PL at 4 h following seeding (Figure 9). Interestingly, the EGMw medium with 1% PL displayed the same degree of efficiency as the EGM-full medium. The supplementation of the EGMw medium with 2.5% and 5% of PL proved to be even more efficient than the EGM-full medium. The addition of PVA–PL nanomats to the EGMw led to the significantly enhanced chemotaxis of the HSVECs compared to both the pure PVA nanomats and the EGMw. Although the proteins released from the PVA–PL nanomats induced the chemotaxis of endothelial cells to a lesser extent than the EGMw medium with PL, only a part of the PL was released from the nanomats at 4 h, as evident from the cumulative release curve (Figure 3). The ability of materials to attract endothelial cells to the wound site is essential for wound healing and angiogenesis.

Vascular repair and re-endothelialization upon injury are complex events in which the proliferation and migration of endothelial cells play a key role. It is known that the VEGF, PDGF, S1P and PD-ECGF present in PL comprise potent chemokines; moreover, a number of other proangiogenic factors, such as FGF-2, HGF and angiopoietin-1 in the PL, are important in terms of their ability to modulate endothelial cell migration. While both the proliferation and migration of endothelial cells in blood vessels are supported by the smooth muscle cells of the contractile phenotype, they are inhibited by the vascular smooth muscle cells of the synthetic phenotype in co-cultures of these two types of cells [45].

#### 3.2.3. The Effect of the PVA Nanomats and PL on 3T3 Fibroblasts

**Figure 10:**
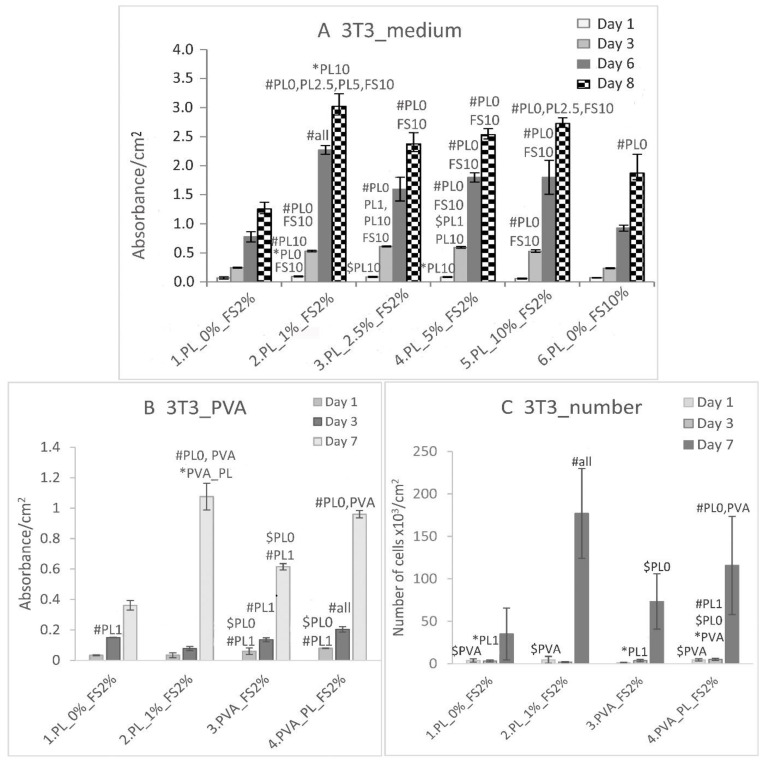
Viability of the 3T3 fibroblasts in the DMEM medium with 2% of FS supplemented with 0, 1, 2.5, 5 and 10% PL, and in the DMEM medium with 10% FS (**A**); viability of the 3T3 cells (**B**) and cell density of the 3T3 cells (**C**) in the DMEM with 2% FS and with 0 and 1% PL, and in the DMEM with 2% FS with PVA and PVA–PL nanomats. Evaluation as performed on days 1, 3, 6 and 8 following seeding (**A**) and on days 1, 3 and 7 following seeding. The data are expressed as the mean ± SD, # refers to *p* < 0.001, $ to *p* < 0.01, * to *p* < 0.05.

On day 1 following seeding, the viability of the 3T3 fibroblasts cultured in a DMEM medium with 2% FS, further supplemented with 0–10% PL, was observed to be higher in the medium with 1% PL than in the medium with 10% PL and the standard DMEM medium with 10% FS (Figure 10A). On day 3, the highest fibroblast viability values were observed in the medium with 2.5–5% PL. On days 6 and 8, the maximum viability was observed in the medium with 1% PL. Thus, the medium with 1% PL and 2% FS was used as the control for further experiments with PVA nanomats. 

With respect to the experiments with the PVA nanomats, the addition of both the PVA and PVA–PL nanomats to the medium acted to support the viability of the 3T3 fibroblasts (Figure 10B) on day 1. At the same time interval, the number of cells was observed to be lowest in the medium with pure PVA (Figure 10C). On day 3, the PVA–PL provided significantly more support for both the viability and proliferation of the 3T3 cells than any of the other samples did (Figure 10B,C). On day 7, the highest viability value was observed in the medium with 1% PL; a lower value, that was still significantly higher than in the medium without PL (PL0%) and pure PVA, was observed in the medium with PVA–PL. The addition of PVA–PL to the medium significantly enhanced the growth of the 3T3 fibroblasts, as evidenced by the occurrence of the highest cell population density in the medium with PVA–PL on day 3 (Figure 10C). On day 7, the highest cell density was observed in the medium with 1% PL, and a lower, but still high cell density was observed in the medium with PVA–PL. Interestingly, pure PVA had a favorable effect on the growth of 3T3 fibroblasts compared to the medium with 2% FS.

An earlier study by Sovkova et al. considered a platelet lysate with a platelet concentration of 800 × 10^9^ platelets/L in various concentrations (2.5–10%) without FBS and with 5% FBS [10]. The fibroblast proliferation was found to be highest in the medium with 7.5–10% PL. Interestingly, the addition of FBS apparently led to an unfavorable outcome. Similarly, in our study, the final number of 3T3 fibroblasts was observed to be higher in the medium with only 2% FS supplemented with PL than in the standard DMEM medium with 10% FS.

**Figure 11:**
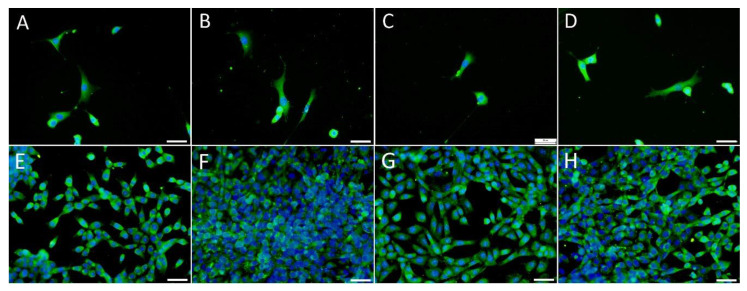
The immunofluorescence staining of vinculin on day 1 following seeding (**A**–**D**) and type I collagen on day 7 following seeding (**E**–**H**) in the 3T3 fibroblasts cultured in the DMEM with 2% FS without PL (**A**,**E**) and with 1% PL (**B**,**F**), the pure PVA nanomats (**C**,**G**) and the PVA–PL nanomats (**D**,**H**). The cell nuclei were counterstained with Hoechst 33258. Olympus IX71 epifluorescence microscope, DP71 digital camera, obj. × 20, scale bar = 50 µm. The same exposure time was used in the taking of the images.

The cell morphology of the fibroblasts was polygonal or spindle-shaped (Figure 11A,D). On day 1, although the cells were positively stained for vinculin, they did not form distinct focal adhesion plaques. On day 7, all the cells were positively stained for collagen I; however, the relatively low cell seeding density and the short culture time interval did not allow for the determination of differences in terms of collagen production between the tested groups of samples. It is known that both the SDF-1 and TGF-β1 released from platelets are responsible for the stimulation of ECM deposition and remodeling [40]. SDF-1 receptors, i.e., CXCR7 and CXCR4, are involved in wound healing. For example, concerning an acute liver injury, SDF-1 was bound to both these receptors in the liver sinusoidal endothelial cells, which acted to stimulate tissue regeneration. However, the chronic injury led to the enhanced expression of the pro-fibrotic CXCR4 receptor, which was further involved in liver fibrosis [46].

Platelet lysate (PL) is one of a number of platelet derivatives that include growth factors and other components capable of influencing the wound healing process. Of the various types of platelet derivatives, platelet-rich plasma (PRP) has been studied in connection with the treatment of chronic wounds without any conclusive results. Although several in vivo studies and clinical trials have been performed aimed at elucidating the therapeutic effect of PRP [2,7,47], its influence on the healing of chronic wounds remains unclear, in part due to the limitations inherent in the randomized controlled trials available. In addition, a higher level of knowledge is required of the specific mechanisms that underlie cellular and tissue functions and the effects on the various cell types involved in wound healing [48]. The potential for the use of platelet derivatives lies in the growth factors and other proteins that are included. This in vitro study considered the use of PL and PVA nanofibers containing PL as potential drug-release membranes for the healing of chronic wounds. The development of skin wound dressings with release factors comprises an interesting approach to skin regeneration [7]. Our results compared the effect of PVA containing PL and three concentrations of PL in a cell culture medium on three differing cell types involved in skin wound healing. In this respect, the results obtained in vitro both reinforced the potential of PL in terms of therapeutic applications and presented a novel biodegradable nanofibrous membrane containing PL as a potential wound healing dressing. 

Nevertheless, since this study presented only an in vitro analysis, further experiments will have to be performed in vivo before the PVA–PL membranes can be considered for clinical trials. Further limitations of the study relate to the exact amount of the PL that is incorporated into the PVA and the biodegradability ratio. These two parameters might be optimized so as to ensure the gradual release of proteins and growth factors and to determine the amounts required to enhance the wound healing process.

## 4. Conclusions

The fabrication of the PVA nanomats with the incorporation of PL as potential wound dressing applications was considered promising due to the numerous beneficial properties of these dressings, including PVA degradability, non-toxicity, nanostructured morphology, the prolonged release of bioactive molecules, the attenuated degradation of bioactive compounds inside the PVA–PL nanomats, and the preservation of the bioactivity of the incorporated PL. In addition, PVA–PL nanomats can be easily produced and stored below −80 °C without any loss of bioactivity. The bioactivity of the PVA–PL nanomats was demonstrated in vitro on HaCaT keratinocytes, endothelial cells (HSVECs) and 3T3 fibroblasts. Moreover, the nanomats acted to support the adhesion, growth and viability of all three cell types, and exerted favorable effects on keratinocytes via the production of basal cytokeratins and the enhanced production of cytokeratin 10, a keratinocyte differentiation marker. The PVA–PL nanomats led to the enhanced chemotaxis of endothelial cells and the production of von Willebrand factor. Since all the cell types considered are involved in the wound healing process, we are convinced that the newly developed PVA–PL nanomats can be considered promising in terms of the treatment of chronic wounds. 

## Data Availability

Data is contained within the present article.

## References

[1] Demidova-Rice T.N., Hamblin M.R., Herman I.M. (2012). Acute and Impaired Wound Healing: Pathophysiology and Current Methods for Drug Delivery, Part 1: Normal and Chronic Wounds: Biology, Causes, and Approaches to Care. Adv. Skin Wound Care.

[2] Martinez-Zapata M.J., Martí-Carvajal A.J., Solà I., Expósito J.A., Bolíbar I., Rodríguez L., Garcia J., Zaror C. (2016). Autologous platelet-rich plasma for treating chronic wounds. Cochrane Database Syst. Rev..

[3] Usui M.L., Mansbridge J.N., Carter W.G., Fujita M., Olerud J.E. (2008). Keratinocyte migration, proliferation, and differentiation in chronic ulcers from patients with diabetes and normal wounds. J. Histochem. Cytochem..

[4] Wang M.J., Qing C., Liao Z.J., Lin W.D., Ge K., Xie T., Shi G.Y., Sheng Z.Y., Lu S.L. (2006). The biological characteristics of dermal fibroblast of the diabetic rats with deep-partial thickness scald. Zhonghua Shao Shang Za Zhi.

[5] Burrow J.W., Koch J.A., Chuang H.H., Zhong W., Dean D.D., Sylvia V.L. (2007). Nitric oxide donors selectively reduce the expression of matrix metalloproteinases-8 and -9 by human diabetic skin fibroblasts. J. Surg. Res..

[6] Rodrigues M., Kosaric N., Bonham C.A., Gurtner G.C. (2019). Wound Healing: A Cellular Perspective. Physiol. Rev..

[7] Pallua N., Wolter T., Markowicz M. (2010). Platelet-rich plasma in burns. Burns.

[8] Italiano J.E., Richardson J.L., Patel-Hett S., Battinelli E., Zaslavsky A., Short S., Ryeom S., Folkman J., Klement G.L. (2008). Angiogenesis is regulated by a novel mechanism: Pro- and antiangiogenic proteins are organized into separate platelet alpha granules and differentially released. Blood.

[9] Vavken P., Sadoghi P., Murray M.M. (2011). The effect of platelet concentrates on graft maturation and graft-bone interface healing in ACL reconstruction in human patients: A systematic review of controlled trials. Arthroscopy.

[10] Sovkova V., Vocetkova K., Rampichova M., Mickova A., Buzgo M., Lukasova V., Dankova J., Filova E., Necas A., Amler E. (2018). Platelet lysate as a serum replacement for skin cell culture on biomimetic PCL nanofibers. Platelets.

[11] Blair P., Flaumenhaft R. (2009). Platelet α-granules: Basic biology and clinical correlates. Blood Rev..

[12] Nurden A.T. (2018). The biology of the platelet with special reference to inflammation, wound healing and immunity. Front. Biosci..

[13] Margraf A., Zarbock A. (2019). Platelets in Inflammation and Resolution. J. Immunol..

[14] Piccin A., Di Pierro A.M., Canzian L., Primerano M., Corvetta D., Negri G., Mazzoleni G., Gastl G., Steurer M., Gentilini I. (2017). Platelet gel: A new therapeutic tool with great potential. Blood Transfus..

[15] Knighton D.R., Ciresi K.F., Fiegel V.D., Austin L.L., Butler E.R. (1986). Classification and treatment of chronic nonhealing wounds. Successful treatment with autologous platelet-derived wound healing factors (PDWHF). Ann. Surg..

[16] Elsaid A., El-Said M., Emile S., Youssef M., Khafagy W., Elshobaky A. (2020). Randomized Controlled Trial on Autologous Platelet-Rich Plasma Versus Saline Dressing in Treatment of Non-healing Diabetic Foot Ulcers. World J. Surg..

[17] Crovetti G., Martinelli G., Issi M., Barone M., Guizzardi M., Campanati B., Moroni M., Carabelli A. (2004). Platelet gel for healing cutaneous chronic wounds. Transfus Apher Sci..

[18] Mohammadi M.H., Molavi B., Mohammadi S., Nikbakht M., Mohammadi M., Mostafaei S., Norooznezhad A.H., Abdegah A.G., Ghavamzadeh A. (2017). Evaluation of wound healing in diabetic foot ulcer using platelet-rich plasma gel: A single-arm clinical trial. Transfus. Apher Sci..

[19] De Angelis B., D’Autilio M.F.L.M., Orlandi F., Pepe G., Garcovich S., Scioli M.G., Orlandi A., Cervelli V., Gentile P. (2019). Wound Healing: In Vitro and In Vivo Evaluation of a Bio-Functionalized Scaffold Based on Hyaluronic Acid and Platelet-Rich Plasma in Chronic Ulcers. J. Clin. Med..

[20] Piccin A., Di Pierro A.M., Tagnin M., Russo C., Fustos R., Corvetta D., Primerano M., Magri E., Conci V., Gentilini I. (2016). Healing of a soft tissue wound of the neck and jaw osteoradionecrosis sing platelet gel. Regen Med..

[21] Ruiz A., Cuestas D., Garcıa P., Jose Quintero J., Forero Y., Galvis I., Velasquez O. (2018). Early intervention in scar management and cutaneous burns with autologous platelet-rich plasma. J. Cosmet. Dermatol..

[22] Klosová H., Stětinský J., Bryjová I., Hledík S., Klein L. (2013). Objective evaluation of the effect of autologous platelet concentrate on post-operative scarring in deep burns. Burns.

[23] Xu F., Zou D., Dai T., Xu H.Y., An R., Liu Y., Liu B. (2018). Effects of incorporation of granule lyophilised platelet-rich fibrin into polyvinyl alcohol hydrogel on wound healing. Sci. Rep..

[24] Miroshnichenko S., Timofeeva V., Permyakova E., Ershov S., Kiryukhantsev-Korneev P., Dvořaková E., Shtansky D.V., Zajíčková L., Solovieva A., Manakhov A. (2019). Plasma-Coated Polycaprolactone Nanofibers with Covalently Bonded Platelet-Rich Plasma Enhance Adhesion and Growth of Human Fibroblasts. Nanomaterials.

[25] Oliveira C., Costa-Pinto A.R., Reis R.L., Martins A., Neves N.M. (2014). Biofunctional nanofibrous substrate comprising immobilized antibodies and selective binding of autologous growth factors. Biomacromolecules.

[26] Blanquer A., Musilkova J., Filova E., Taborska J., Brynda E., Riedel T., Klapstova A., Jencova V., Mullerova J., Kuzelova Kostakova E. (2021). The Effect of a Polyester Nanofibrous Membrane with a Fibrin-Platelet Lysate Coating on Keratinocytes and Endothelial Cells in a Co-Culture Systém. Nanomaterials.

[27] Adamson A.W., Gast A.P. (1997). Physical Chemistry of Surfaces.

[28] Patnaik A., Rengasamy R.S., Kothari V.K., Ghosh A. (2006). Wetting and Wicking in Fibrous Materials. Textile Progress.

[29] Halima N.B. (2016). Poly(vinyl alcohol): Review of its promising applications and insights into biodegradation. RSC Adv..

[30] Kamoun E.A., Chen X., Mohy Eldin M.S., Kenawy E.-R.S. (2015). Crosslinked poly(vinyl alcohol) hydrogels for wound dressing applications: A review of remarkably blended polymers. Arab. J. Chem..

[31] Zhang X., Tang K., Zheng X. (2016). Electrospinning and crosslinking of COL/PVA Nanofiber-microsphere Containing Salicylic Acid for Drug Delivery. J. Bionic Eng..

[32] Alves M.-H., Jensen B.E.B., Smith A.A.A., Zelikin A.N. (2011). Poly(Vinyl Alcohol) Physical Hydrogels: New Vista on a Long Serving. Biomater. Macromol. Biosci..

[33] Koprivova B., Lisnenko M., Solarska-Sciuk K., Prochazkova R., Novotny V., Mullerova J., Mikes P., Jencova V. (2020). Large-scale electrospinning of poly (vinylalcohol) nanofibers incorporated with platelet-derived growth factors. Express Polym. Lett..

[34] Rampichová M., Buzgo M., Míčková A., Vocetková K., Sovková V., Lukášová V., Filová E., Rustichelli F., Amler E. (2017). Platelet-functionalized three-dimensional poly-ε-caprolactone fibrous scaffold prepared using centrifugal spinning for delivery of growth factors. Int. J. Nanomed..

[35] Baik S.Y., Lim Y.A., Kang S.J., Ahn S.H., Lee W.G., Kim C.H. (2014). Effects of Platelet Lysate Preparations on the Proliferation of HaCaT Cells. Ann. Lab. Med..

[36] Barsotti M.C., Losi P., Briganti E., Sanguinetti E., Magera A., Al Kayal T., Feriani R., Di Stefano R., Soldani G. (2013). Effect of platelet lysate on human cells involved in different phases of wound healing. PLoS ONE.

[37] Schoop V.M., Mirancea N., Fusenig N.E. (1999). Epidermal Organization and Differentiation of HaCaT Keratinocytes in Organotypic Coculture with Human Dermal Fibroblasts. J. Investig. Dermatol..

[38] El-Ghalbzouri A., Gibbs S., Lamme E., van Blitterswijk C.A., Ponec M. (2002). Cutaneous Biology. Effect of fibroblasts on epidermal regeneration. Br. J. Dermatol..

[39] Oliveira S.M., Pirraco R.P., Marques A.P., Santo V.E., Gomes M.E., Reis R.L., Mano J.F. (2016). Platelet lysate-based pro-angiogenic nanocoatings. Acta Biomater..

[40] Eisinger F., Patzelt J., Langer H.F. (2018). The Platelet Response to Tissue Injury. Front. Med..

[41] Romaldini A., Ulivi V., Nardini M., Mastrogiacomo M., Cancedda R., Descalzi F. (2019). Platelet lysate inhibits NF- κB activation and induces proliferation and an alert state in quiescent human umbilical vein endothelial cells retaining their differentiation capability. Cells.

[42] Muraglia A., Todeschi M.R., Papait A., Poggi A., Spanò R., Strada P., Cancedda R., Mastrogiacomo M. (2015). Combined platelet and plasma derivatives enhance proliferation of stem/progenitor cells maintaining their differentiation potential. Cytotherapy.

[43] Moghadam F.H., Tayebi T., Moradi A., Nadri H., Barzegar K., Eslami G. (2014). Treatment with platelet lysate induces endothelial differentiation of bone marow mesenchymal stem cells under fluid shear stress. EXCLI J..

[44] Lenting P.J., Christophe O.D., Denis C.V. (2015). Von Willebrand factor biosynthesis, secretion, and clearance: Connecting the far ends. Blood.

[45] Tang R., Zhang G., Chen S.-Y. (2016). Smooth muscle cell proangiogenic phenotype induced by cyclopentenyl cytosine promotes endothelial cell proliferation and migration. J. Biol. Chem..

[46] Ding B.-S., Cao Z., Lis R., Nolan D.J., Guo P., Simons M., Penfold M.E., Shido K., Rabbany S.Y., Rafii S. (2014). Divergent angiocrine signals from vascular niche balance liver regeneration and fibrosis. Nature.

[47] Yang H.S., Shin J., Bhang S.H., Shin J.Y., Park J., Im G.I., Kim C.S., Kim B.S. (2011). Enhanced skin wound healing by a sustained release of growth factors contained in platelet-rich plasma. Exp. Mol. Med..

[48] Borzini P., Mazzucco L. (2005). Platelet gels and releasates. Curr. Opin. Hematol..

